# Analyses of canine cancer mutations and treatment outcomes using real-world clinico-genomics data of 2119 dogs

**DOI:** 10.1038/s41698-023-00346-3

**Published:** 2023-01-19

**Authors:** Kevin Wu, Lucas Rodrigues, Gerald Post, Garrett Harvey, Michelle White, Aubrey Miller, Lindsay Lambert, Benjamin Lewis, Christina Lopes, James Zou

**Affiliations:** 1 One Health Company, Palo Alto, CA US; 2 Department of Biomedical Data Science, Stanford University, Stanford, US

**Keywords:** Cancer models, Cancer genomics, Cancer genomics

## Abstract

Spontaneous tumors in canines share significant genetic and histological similarities with human tumors, positioning them as valuable models to guide drug development. However, current translational studies have limited real world evidence as cancer outcomes are dispersed across veterinary clinics and genomic tests are rarely performed on dogs. In this study, we aim to expand the value of canine models by systematically characterizing genetic mutations in tumors and their response to targeted treatments. In total, we collect and analyze survival outcomes for 2119 tumor-bearing dogs and the prognostic effect of genomic alterations in a subset of 1108 dogs. Our analysis identifies prognostic concordance between canines and humans in several key oncogenes, including *TP53* and *PIK3CA*. We also find that several targeted treatments designed for humans are associated with a positive prognosis when used to treat canine tumors with specific genomic alterations, underscoring the value of canine models in advancing drug discovery for personalized oncology.

## Introduction

Despite a growing number of targeted cancer treatments in development each year, few are able to reach cancer patients due to the rate at which evidence is generated from human clinical trials^1,2^. As such, complementary models for cancer are necessary to effectively move from preclinical investigations to clinical translation. In particular, spontaneous tumors in canines are valuable sources of evidence for understanding human cancers, with one in three dogs developing cancer within their lifetime^3,4^. Dogs and humans share similar environments, nutrition, intact immune systems, cancer histology, therapeutic response, acquired resistance, recurrence, metastasis, and genetic and molecular targets^5–11^.

There is a growing body of evidence that supports the study of canine cancers for translational and clinical research^8,11–15^. Comparative oncology studies between canines and humans have increased dramatically over the last few years, as the interrogation into the genomic characteristics of cancer in dogs has expanded. In the canine genome, 19,000 genes have been identified that are orthologous to genes in the human genome^16,17^. Canine DNA and protein sequences are more similar than mice to humans, with dogs sharing over 650 Mb of ancestral sequence with humans that are not shared in rodents^11,16,18^. DNA sequencing sheds light on prevalent genomic mutations and highlights the close biological and molecular similarities between canine and human cancers^19^. Indeed, hundreds of similar genetic variations and somatic driver mutations between human and canine cancer have been reported^13,14,17,20^. Genomic analyses of canine tumors have identified and provided insights into the role of oncogenes and tumor suppressor genes of specific tumors in humans^21^. However, there is still a lack of knowledge on the prognosis and effect of these gene alterations and responses to treatment.

The last few decades have seen a significant increase in the attention and resources for treating canine cancers in veterinary clinics, providing a unique opportunity to study the prognostic effects of targeted therapies^22^. In addition, next-generation sequencing (NGS) in canine tumors has identified shared biomarkers between human and canine cancers, leading to a shift in individualized cancer treatments in canines^23^. New small molecule-targeted therapies are constantly under development to improve cancer care in humans, but this process is often very expensive and prolonged, with a high failure rate of 89% of novel drugs in clinical trials^1,2^. Clinico-genomic data from canine tumors can be used to identify signals from therapeutic responses to inform and guide drug development in humans. Statistical learning methods have previously been applied to large-scale human clinico-genomic data to predict treatment outcomes using the molecular profile of cancer to prognosis^24^. In this study, we apply similar predictive modeling to real-world clinical-genomic data from the FidoCure® Precision Medicine Platform to identify biomarkers associated with prognosis and treatment prediction. Our work underscores the promise of spontaneous models of cancer in canines in helping realize the potential of precision medicine in humans.

## Results

### Data summary

**Fig. 1:**
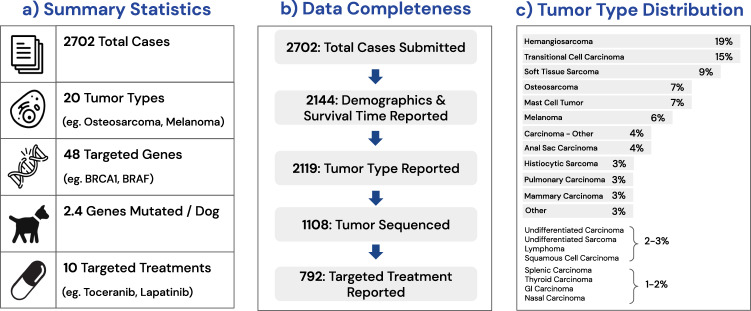
Summary of the dataset used for analysis. **a** Counts of the unique number of cases, tumor types, targeted genes, mutations/dog, and targeted treatments. **b** The number of dogs with complete values for each feature reported. Completeness is reported cumulatively at each step. For example, the 2119 cases with complete tumor type reported also have complete demographics and survival times. **c** The distribution of tumor types in dogs, as a percentage of the 2119 dogs with tumor types, demographics, and survival outcomes reported.

Of the 2702 total cases collected, 2144 contained demographic information and survival time from diagnosis. Among this group, 2119 were annotated with the tumor types, which were divided into 19 different histological categories (e.g., soft tissue sarcoma). A further filtering of these cases based on the ability to produce high-quality DNA NGS yielded a total of 1108 dogs. Of these dogs, a total of 792 cases also contain targeted treatment information. In total, mutations in 48 targeted genes were identified, with an average of 2.4 mutated genes per dog. This group of dogs, which also had demographic and survival outcomes, was treated with 10 targeted therapies (Fig. 1a, b).

### Tumor types

Tumor types were divided into 19 broad categories (in order of prevalence): *hemangiosarcoma, transitional cell carcinoma, soft tissue sarcoma, osteosarcoma, mast cell tumor, melanoma, carcinoma (other), anal sac carcinoma, histiocytic sarcoma, pulmonary carcinoma, mammary carcinoma, undifferentiated carcinoma, undifferentiated sarcoma, lymphoma, squamous cell carcinoma, splenic carcinoma, thyroid carcinoma, GI carcinoma, nasal carcinoma*, with the most common being hemangiosarcomas (~19%). Meanwhile, transitional cell carcinomas and soft tissue sarcomas are represented in ~15% and ~9% of dogs, respectively. Figure 1c presents the overall distribution of tumors as divided into 19 types and an additional ‘other’ category.

The hazard ratios of each tumor type relative to the other tumor types were reported in the panel. To model this effect, one tumor type covariate was included at a time and represented as an indicator variable (indexed in the equation below as *j*). The dog’s weight, sex, reproductive status, age at diagnosis, and time from diagnosis to treatment were used as control variables. The model is defined as follows:

1 $$\begin{array}{l}Survival\,from\,Diagnosis\sim Tumor\,Type_j \ast \beta _1 + Weight \ast \beta _2 + Sex \ast \beta _3\\ \quad+ \,Reproductive\,Status \ast \beta _4 + Age\,at\,Diagnosis \ast \beta _5\\ \quad+ \,Time\,from\,Diagnosis\,to\,Treatment \ast \beta _6\end{array}$$

The results from this analysis are found in Table 1, which includes the hazard ratios associated with each tumor type observed in the dataset. We have also included the Kaplan–Meier curves for patients with and without each tumor type in Supplementary Fig. 3, and find that they are consistent for tumor types with the largest and smallest effect sizes, with as hemangiosarcomas, GI carcinomas, anal sac carcinomas, thyroid carcinomas, and soft tissue sarcomas. The Kaplan–Meier estimate of median survival time (in days) for patients with each tumor type, as well as patients without (control) were also computed. Of the 19 total tumor groups, 9 tumor types had hazard ratios that were statistically significant when controlling for other covariates used in the model. Of these nine, hemangiosarcomas had the highest hazard ratio of 2.07, with a median survival time of 203 days (*P* < 0.01), compared to a median survival time of 421 in the control group, followed by GI carcinoma and histiocytic sarcoma with a median survival time of 204 and 211 days (OS HR = 1.96, *P* = 0.01; OS HR = 1.55, *P* = 0.01) and 370 and 373 days of controls, respectively. Oppositely, thyroid carcinoma had the lowest hazard ratio of 0.35, with a median survival time of 1007 days, compared to 365 days in the control group.

**Table 1:** Statistically significant hazard ratios of tumor type on survival from diagnosis.

Hazard ratios associated with tumor type				
Tumor type	Hazard ratio (95% CI)	*p*-value	Sample size (Tumor type/others)	Median survival days (Tumor type/others)
Hemangiosarcoma	2.07 (1.79,2.38)	<0.01	405/1714	203/421
GI carcinoma	1.96 (1.15, 3.34)	0.01	25/2094	204/370
Histiocytic sarcoma	1.55 (1.14, 2.10)	0.01	73/2046	211/373
Lymphoma	1.47 (1.08, 1.99)	0.01	59/2060	298/372
Osteosarcoma	1.40 (1.10, 1.79)	0.01	152/1967	256/377
Melanoma	0.74 (0.57, 0.97)	0.03	117/2002	379/366
Soft tissue sarcoma	0.64 (0.52, 0.79)	<0.01	220/1899	506/353
Anal sac carcinoma	0.39 (0.27, 0.55)	<0.01	87/2032	812/349
Thyroid carcinoma	0.35 (0.17, 0.69)	<0.01	28/2091	1007/365

Hazard ratios are presented with 95% confidence intervals, and only tumor types with *p*-values < 0.05 are included. Sample sizes and median survival time (in days) are reported for dogs with and without each tumor type.

### Hazard ratios for gene mutations

**Fig. 2:**
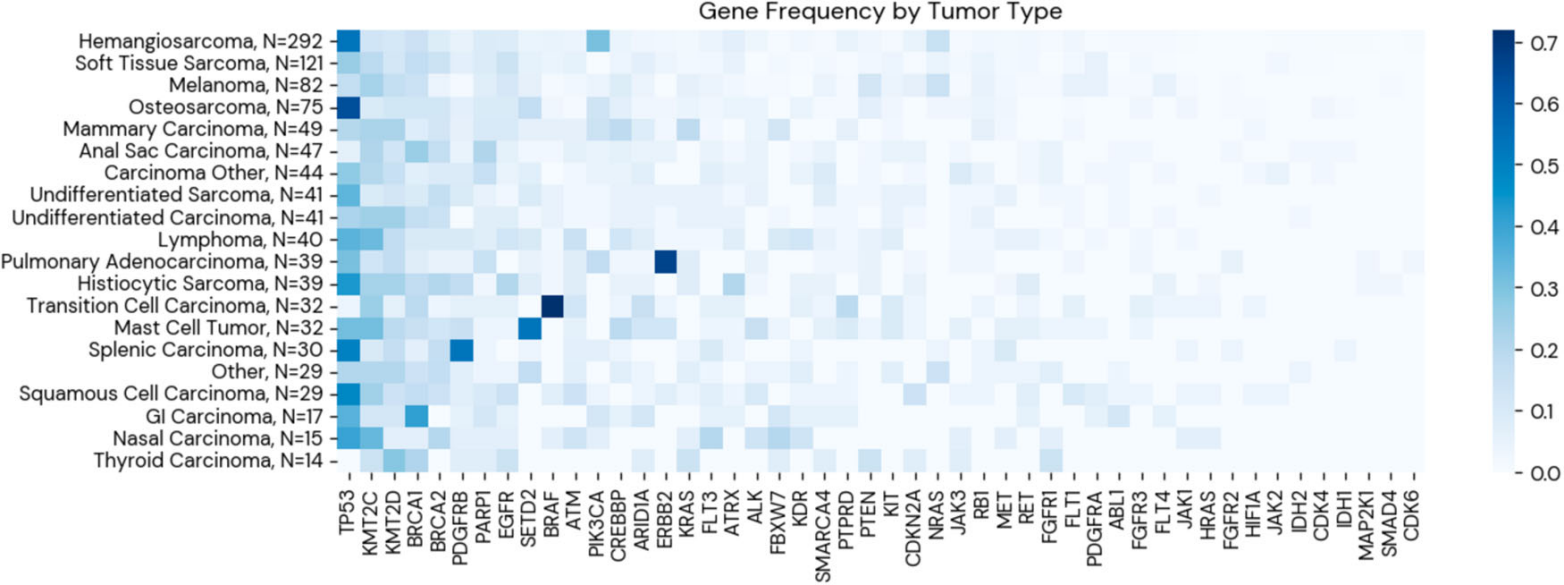
Frequency of Gene Mutations by Tumor Type. For each tumor type (row), the relative frequency of tumors that contain a given mutation is contained in each column value. For example, a value of 1 would mean that all instances of a given tumor type contain a certain mutation. The sample size for each tumor reflects the number of cases with reported genetic data.

The effect of somatic mutations in targeted genes on overall survival rates was analyzed, including the coding exons of 48 genes commonly mutated in human cancers. To preserve sufficient sample size, mutations were grouped at the gene level. We report the distribution of targeted gene mutations in each tumor type in Fig. 2. In our model, a targeted gene is encoded as mutated if it contains one or more somatic mutations. To model the effect of mutations in a targeted gene on overall survival, the presence of a mutation in a given gene (indexed in the equation below using *j*) as a binary variable was included. In addition, the dog’s weight, sex, reproductive status, age at diagnosis, time from diagnosis to treatment, and tumor type were included as control variables. The Cox proportional-hazards model was used, defined as follows:

2 $$\begin{array}{l}Survival\,from\,Diagnosis\sim Gene_j \ast \beta _1 + Weight \ast \beta _2 + Sex \ast \beta _3\\ \quad+ \,Reproductive\,Status \ast \beta _4 + Age\,at\,Diagnosis \ast \beta _5\\ \quad+ \,Time\,from\,Diagnosis\,to\,Treatment \ast \beta _6\\ \quad+ \,Tumor\,Type_1 \ast \beta _7 + ... + Tumor\,Type_{19} \ast \beta _{25}\end{array}$$

Results from this model are presented in Table 2. Mutations in five targeted genes, *TP53*, *PIK3CA*, *NRAS, ATM*, and *KIT*, were associated with statistically significant hazard ratios of 1.48, 1.33, 0.60, 0.51, and 0.44, respectively. *TP53* and *PIK3CA* were associated with higher risk, with median survival times of 220 and 189 days, while the median survival times of the control groups were 420 and 370 days. *KIT* was found to be associated with relatively lower risk, with a median survival time of 642 days compared to 344 days in the control group. Additionally, we have included Kaplan–Meier curves for gene mutations as well as treatment outcomes (Supplementary Fig. 2). These plots provide 95% confidence intervals for both cohorts across days since diagnosis. We find that the Kaplan–Meier curves are generally consistent with the statistical significance reported in Table 2.

**Table 2:** Statistically significant hazard ratios associated with mutations in each gene, confidence intervals, *p*-values, sample sizes, and median survival in days.

Hazard ratios associated with gene mutations				
Mutated gene	Hazard ratio (95% CI)	*p*-value	Sample size (with/without)	Median survival days (with/without)
*TP53*	1.48 (1.24, 1.77)	<0.01	379/711	220/420
*PIK3CA*	1.33 (1.03, 1.72)	0.03	129/979	189/370
*NRAS*	0.60 (0.41, 0.88)	<0.01	69/1039	342/350
*ATM*	0.51 (0.32, 0.79)	0.01	58/1050	809/343
*KIT*	0.43 (0.21, 0.88)	0.02	31/1077	642/344

Hazard ratios are presented with 95% confidence intervals, and only targeted genes with *p*-values < 0.05 are included. Sample sizes and median survival time (in days) are reported for dogs both with and without mutations in each gene.

### Treatment effects for each targeted gene

We analyze the prognostic effects of treatments conditioned on each tumor’s genomic alterations. The co-occurrence of treatments ordered for patients is described in Supplementary Fig. 1. To model an individual patient’s response to treatment, we used the subset of the data which includes all dogs with a given mutation. Within this subset, the survival rates of patients given each drug were observed. The dog’s weight, sex, reproductive status, tumor type, age at diagnosis, and time from diagnosis to treatment were used as control variables. Treatment and tumor type are one-hot encoded and represented in the model below with 10 treatments and 19 tumor types. The model is defined as follows:

3 $$\begin{array}{l}Survival\,from\,Treatment\sim Weight \ast \beta _1 + Sex \ast \beta _2 \\\quad+\, Reproductive\,Status \ast \beta _3\\ \quad+ \,Age\,at\,Diagnosis \ast \beta _4 + Time\,from\,Diagnosis\,to\,Treatment \ast \beta _5\\ \quad+ \,Treatment_1 \ast \beta _6 + ... + Treatment_{10} \ast \beta _{15}\\ \quad+ \,Tumor\,Type_1 \ast \beta _{16} + ... + Tumor\,Type_{19} \ast \beta _{34}\end{array}$$

During training, covariates with high correlation were removed due to multicollinearity. Additionally, genes that did not have a large enough sample size in patients were not included (i.e., cases where *p* > *n*). Furthermore, we analyzed the association of functional pathways of gene mutations, but did not find additional statistically significant drug responses associated with gene families and common pathways. In general, we find that grouping low-occurrence genomic alterations into functional pathways does not improve their statistical signal. Somatic variants of each gene were analyzed and germline variants were excluded according to a previous publication^17^. The results from our analysis of treatment responses are found in Table 3. We find four gene-drug combinations with hazard ratios less than one and with p-values less than 0.05. Each row of Table 3 represents the effect of treatment in the subpopulation which has at least one mutation in a given targeted gene. *BRAF* mutant tumors had a better prognosis when treated with lapatinib (EGFR and ERBB2 inhibitor) (OS HR 0.10, P = 0.02) and *ARID1A* with trametinib (MEK1/2 inhibitor) (OS HR 0.08, P = 0.02). Differently from other genes, BRCA1 and BRCA2 germline and somatic mutations were included in the analysis. Since both can be targetable, we just excluded neutral variants evaluated by the PROVEAN tool. Protein Variation Effect Analyzer (PROVEAN) is a software used to predict an amino acid substitution or indel impact on the biological function of a protein^25^. BRCA1 unknowns and deleterious mutations were correlated with better prognosis in dogs treated with dasatinib (OS HR 0.26, P = 0.02). Additionally, we include the results from an analysis including both germline and somatic variants, contained in Supplementary Table 3. While 33% of amino acid changes in our dataset are classified as germline, their inclusion only increases our overall patient sample size by 6.5%. We find the results are mostly in-line with our previous analysis that excludes germline mutations (with only small deviations in hazard ratios). Notably, we find that patients with ALK mutations have a median survival of 424 days compared to 349 days in the group without ALK mutations, for an overall survival hazard ratio of 0.64 (0.42, 0.97) and p-value of 0.04. These results indicate that germline mutations play a minor role in assessing overall risk in our set of oncogenes.

**Table 3:** Hazard ratios associated with treatment and gene combination, confidence intervals, *p*-values, sample sizes, and MST.

Hazard ratios associated with treatment and gene mutation combinations					
Targeted gene	Treatment	Hazard ratio (95% CI)	*p*-value	Sample size (Treatment/control)	Median survival days (Treatment/control)
BRCA1	Dasatinib	0.26 (0.08, 0.82)	0.02	11/85	284/183
BRAF	Lapatinib	0.10 (0.02, 0.65)	0.02	24/21	641/202
ARID1A	Trametinib	0.08 (0.01, 0.69)	0.02	18/20	237/185

Hazard ratios are presented with 95% confidence intervals, and only treatment-gene combinations with *p*-values < 0.05 are included. Sample sizes and median survival time (in days) are reported for dogs in the treatment and control groups.

## Discussion

In this study, we analyzed the clinico-genomic data of 1108 dogs with spontaneous tumors from 2702 dogs enrolled in the FidoCure® Personalized Platform. Single nucleotide variants and indels of exosome sequences of 48 commonly mutated genes were evaluated in over 19 canine tumor types collected across the US. From these dogs, treatment outcome data of 10 small molecule target therapies were also collected. Data used in this analysis was provided by the FidoCure® Personalized Platform which combines genomic information from dogs naturally affected by tumors, demographics, clinical characteristics, and treatment outcomes.

Over the last several years, progress has been made to better characterize the genome of canine tumors^13,15,17,21,26^, but the connection between genetic profiles to prognosis and therapeutic response is still unclear. The overlap of human and canine mutations associated with the therapeutic response using real-time data from pet dogs being treated for cancer can accelerate new drug development and help define novel strategies to treat tumors in humans^17^. Several research groups specialized in genomics have recognized the benefits of dogs in comparative analysis and understanding of the human genome^27^. At the same time, the virtuous cycle of information from human to canine oncology can bring new treatment options to dogs with cancer. The analysis performed in this study underscores the importance of the spontaneous dog model of cancer to the comparative oncology field by demonstrating the ability to perform precision targeting of somatic events for specific therapeutic interventions.

First, to better understand the concordance of these results to human tumors, we analyzed data across 10 pan-cancer human studies^28^ and reported hazard ratios and statistical significance in the same manner as canine tumors are presented (Supplementary Table 1). We find concordance in the relative risk of TP53, ATM, and KIT mutations in canines and humans, while PIK3CA and NRAS mutations present different risks.

To understand the divergence in PIK3CA and NRAS, we analyzed the underlying tumor distributions in our dataset. Canine spontaneous hemangiosarcoma is a useful model for angiosarcoma both in their histologies and common driver mutations, including NRAS, PLCG1, PIK3CA, and TP53^21^. In previous work, we identified that PIK3CA and NRAS mutations were both enriched with hemangiosarcoma (*p* = 1.88 × 10^–7^ and *p* = 2.27 × 10^–5^, respectively)^17^. Angiosarcoma is considered a rare neoplasm in humans but hemangiosarcoma is very common in dogs, representing 19% of tumors analyzed in this study. We attribute the difference in the overall survival of PIK3CA and NRAS between dogs and humans to the high frequency of hemangiosarcoma analyzed in this study, representing 19% of studied dogs. Hemangiosarcoma presents with the highest risk among tumor types in our panel (HR 2.07, p < 0.01).

Our analysis captures the association of *TP53* mutations in canine tumors with a worse prognosis compared to other mutations, after controlling for factors such as demographics and tumor type. These results are in concordance with human-specific tumors such as non-small cell lung cancer, metastatic breast cancer, and pancreatic carcinoma^29–32^. Similar to humans, *TP53* mutations in canines are usually localized in the DNA-binding domain and are frequently associated with a loss of normal function resulting in uncontrolled cell proliferation^26,33^. *TP53* is the most common mutated gene in both human tumors^34^ and dogs^17,26^. Although pulmonary carcinoma, mammary tumors, and pancreatic carcinomas are not over-represented in our canine-data set, *TP53* is mutated in more than 25% of osteosarcoma in both species^26^, which represents 7% of tumor subtypes in this analysis. In human osteosarcoma, *TP53* mutations have been correlated with a negative impact on 2-year overall survival in a meta-analysis of 8 published studies when compared to wild types^35^.

In our canine-data set, hemangiosarcoma represents the most common tumor type, comprising 19% of all tumors. In accordance with previous studies, this tumor type has a high frequency of *TP53* mutations^17,21^, which has a relatively poor prognosis compared to other mutations (OS HR 1.48, *N* = 379, *P* < 0.01). Hemangiosarcoma shares its high rate of *PIK3CA* mutations with angiosarcoma, a rare tumor with poor survival rates in humans^13,21^. We observe that PIK3CA has a relatively high hazard ratio (OS HR 1.33, *N* = 129, *P* = 0.03), suggesting its association with a worse prognosis in dogs with cancer.

Our results indicate favorable clinical outcomes of *NRAS (*OS HR 0.60, *N* = 69, *P* < 0.01)*, ATM (*OS HR 0.51, *N* = 58, *P* = 0.01), and *KIT* mutations (OS HR 0.43, *N* = 31, *P* = 0.02). NRAS is a commonly mutated oncogene in human cancer, the majority of mutations are localized in codons 12, 13, and 61. This gene is altered in 3.03% of all cancers and has a higher prevalence in melanoma, colon carcinoma, and acute myeloid leukemia (https://www.mycancergenome.org). In melanoma, NRAS mutations occur in 15 to 23.15% of tumors and are usually mutually exclusive with BRAF and KIT^36^. Although NRAS mutation has been associated with shorter survival time for patients with metastatic melanoma^37^, NRAS mutation does not have a prognostic impact on acute myelogenous leukemia^38^. Studies using MEK inhibitors in patients with NRAS mutation are currently under investigation^39^.

ATM gene encodes a serine-threonine kinase essential in the detection and signaling to repair DNA double-strand breaks and is commonly mutated in a variety of human cancers, including mantle cell lymphoma, colorectal, lung, prostate, pancreatic, and other^40^. ATM is mutated in approximately 5% of cancers and this incidence can be higher as 40% in mantle cell lymphoma^41^. The majority of these alterations are missense mutations scattered throughout the coding region, and there are a few hotspots such as R377C/H. ATM mutations commonly induce protein truncation and destabilization resulting in loss of protein function^42^. ATM mutations in patients with metastatic colorectal tumors are associated with better prognosis^43^. Tumors with ATM mutation are candidates for PARP inhibitor treatment^44^.

In humans, most *KIT* mutations are deleterious in exon 11 and frequently span critical codons 557 and 588^45,46^. In our panel, KIT mutations are distributed across all 20 tumor types, with the three most common being soft tissue sarcomas (15.3%), melanomas (9.8%), and osteosarcomas (8.9%) (total *N* = 885). On the other hand, in a study of 1637 KIT mutations in humans, the three most common tumor types are GI stromal tumors (30.9%), melanomas (9.5%), and non-small cell lung cancers (9.4%)^47^. Mutations occurring in *KIT* proto-oncogene receptor tyrosine kinase are considered the major molecular drivers of most gastrointestinal stromal tumors (GIST) in people^48^. Previous publications report *KIT* mutations to be commonly associated with poor prognosis in human gastrointestinal stromal tumors^49,50^. To account for the differing tumor type distributions between dogs and humans, we analyzed the risk of KIT mutations across all tumor types using a human pan-cancer database of 1218 KIT mutations (and 34195 control cases)^28^. We find that KIT mutations have a hazard ratio of 0.73 (0.66, 0.81), *p* < 0.01, which is comparable to our finding of better outcomes in dogs (HR = 0.43 (0.21, 0.88), *p* = 0.02).

Previous work has found that human tumors carrying *KIT* mutations have a better clinical response when treated with a tyrosine kinase inhibitor^51^. However, we do not find such treatments statistically significant in our study. Among patients with advanced gastrointestinal stromal tumors treated with imatinib, the specific genotype is the major prognostic factor, as the presence of exon 9-activating mutations is related to longer progression-free survival compared to exon 11-mutations^52,53^. *KIT* mutations in canine mast cell tumors have been associated with increased recurrence and death^54^ and did not predict treatment response to toceranib treatment, a tyrosine kinase inhibitor^55^. *KIT* mutation status is correlated with a good prognosis in tumors in dogs, and further studies are needed to be performed to better understand this signal.

We also find that somatic variants of *BRAF* are correlated with a positive prognosis given lapatinib treatment. Lapatinib is a kinase inhibitor of the intracellular tyrosine kinase domains of both epidermal growth factor receptor (HER1/EGFR/ERBB1) and human epidermal growth factor receptor type 2 (HER2/ERBB2). Interestingly, *EGFR* inhibitors have been identified to have an anti-cancer effect in cancer cells carrying *BRAF* mutation. In a previous publication, afatinib (an epidermal growth factor receptor inhibitor) effectively decreased the viability of *BRAF*^V600E^ mutant colon cancer either in *EGFR*-wildtype or *EGFR*-mutated cells^56^. Additionally, it is well known that *BRAF* mutations lead to the constitutive activation of EGFR downstream signaling pathway bypassing EGFR blockage reducing the target therapy action of EGFR inhibitors. The use of anti-EGFR molecules has contributed to improved outcomes for patients carrying *BRAF*^V600E^ mutations^57–60^. The same effect reported in these studies could potentially explain the positive prognosis of dogs carrying *BRAF* mutation when receiving lapatinib. On the other hand, we do not observe statistically significance effects with MEK inhibitor trametinib or PARP inhibitor olaparib due to a lack of sample size in either the treatment or control groups.

*ARID1A* is a subunit of the SWI/SNF chromatin remodeling complex that plays a role in altering chromatin structure for transcription, DNA synthesis, and DNA repair^61^. This tumor suppressor gene also has a positive role downstream in the MEK/ERK pathway demonstrated in the human colon carcinoma cell line^62^. *ARID1A* is indeed mainly localized as enhancers of tumor cells acting as a cofactor at regions bound by AP1 transcription factors, which act downstream in the MEK/ERK pathway^62,63^. In KRAS-mutated colorectal cancers, trametinib leads to the attenuation of MEK/ERK pathways, similar to the effect of *ARID1A* loss in these cells^62^. The dual effect of trametinib treatment and *ARID1A* loss on the MEK-ERK pathway could be related to the better prognosis seen in dogs with *ARID1A* mutation when treated with MEK inhibitor and suggests further investigation. Currently, tumors carrying *ARID1A* mutations can be treated with tazemetostat, a histone methyltransferase EZH2. In humans, we also find higher sensitivity of mutated ARID1A when treated with trametinib in lung (log2-sens −0.700/−0.307), ovarian (log2-sens −0.513/−0.159), and brain (log2-sens −0.088/0.069) tumors compared to wild type cell lines (Dependency Map Portal (Depmap) for PRISM Repurposing Primary Screen 19Q4)^64^. The correlation found in this analysis allows us to hypothesize future studies to investigate the potential benefits of MEK-ERK pathway inhibition for tumors carrying *ARID1A* mutation.

*BRCA1* somatic and germline mutations classified as deleterious and unknown using PROVEAN tools were correlated with better prognosis when treated with dasatinib. Loss-of-function mutations in *BRCA1* not only increase the risk of cancer development in humans and dogs but also increase the genomic stability of cells^65–67^. *BRCA1* is a critical double-strand break repair that utilizes homologous recombination, cell cycle regulation, and protein ubiquitination^68,69^. Homologous recombination occurs primarily during the G2 and S cell cycle phase and leads to double-strand break repair^69^. Treatment with platinum-based chemotherapy and PARP inhibitors is usually recommended in humans carrying *BRCA1* and *BRCA2* mutations. In humans, lung (log2-sens −1.68 vs −2.44), colon (log2-sens −0.98 vs −1.43), and ovarian (log2-sens −2.322 vs −2.325) cancer cell lines carrying BRCA1 mutation have higher sensibility to dasatinib compared to cell lines without BRCA1 mutation^70^.

Breast cancer triple-negative basal-like is a tumor subtype in humans known to have a lack of molecular targets and aggressive biological behavior^71^. Preclinical studies have demonstrated a more potent anticancer effect with dasatinib, a multiple tyrosine kinase inhibitor, in triple-negative basal-like cells when compared to other breast cancer subtypes^72^. In particular, dasatinib showed prognostic benefits in human patients with advanced and refractory triple-negative metastatic breast cancer, as opposed to other tumor subtypes^73^. This specific subtype also has dysfunction, loss of activity, lower levels of *BRCA1*, and more sensitivity to DNA-damage agents such as doxorubicin and cisplatin^74–76^. When used to treat *BRCA1* mutated cells, dasatinib alone inhibits Chk1, inducing DNA damage and arresting cells in G1^77^ that have a dysregulation of homologous recombination during the G2/S cell phase. The outcomes observed in canine tumors suggest that the relationship between dasatinib and *BRCA1* mutated cells should be further investigated in humans.

An important translational gap also exists in dosages and toxicity in the targeted therapies used in both canines and humans. To this end, all compounds evaluated in this analysis have been published as components of regulatory submissions to the U.S. Food and Drug Administration (FDA). These canine studies evaluate the toxicity effect of each compound as well as the plasma kinetics (area under the curve, concentration and time) and also ADME (absorption, distribution, metabolism, and excretion). Similar pharmacokinetics and toxicities effects have been identified in both species. In addition, these compounds have been used in clinical trials and in vitro studies for canine tumors. To aid in understanding the differences of dosage in translating canine and human treatment outcomes, we have included the recommended dosages for each targeted treatment used in our study, along with the human dosages (Supplementary Table 5).

These systematic analyses allow for comparisons to be made between human and canine cancer genomic studies. A limitation of our canine pan-cancer analysis is the difference in tumor type distributions compared to human pan-cancer studies. For example, breast, prostatic, and lung cancers have lower relative prevalence in canines compared to humans, while angiosarcoma/hemangiosarcoma and urothelial carcinomas are overrepresented in canines compared to humans. Due to the sample size limitations of this study, we are unable to report tumor type-gene mutation interactions with statistical significance. As the reported risks associated with gene mutations are highly correlated to the severity of different tumor types, a follow-on study focused on such interactions would further improve our understanding of concordance between canine and human models of cancer. On the other hand, canine models are advantageous for tumor types that are rare in humans but common in dogs. Canine tumors have been used as a powerful platform for translation investigation specially due to close biological and molecular similarities between several canine and humans tumors such as lymphoma^78,79^, osteosarcoma^15,80^, hemangiosarcoma^13,21,81^, glioma^82^, melanoma^83^, mammary tumors^14^, and urothelial carcinoma^84^. Biological and genetic information gathered from canines could serve to guide hypothesis generation given limited human clinical data.

Our findings lay the groundwork for using real-world data to quantify the treatment response of small molecule therapies on canine patients with mutations in specific oncogenes. Using the largest clinico-genomic canine cancer dataset to date, we integrate outcomes data with targeted therapies and tumor profiles to aid in hypothesis generation in cancer drug discovery.

## Materials and methods

### Dog enrollment and sample collection

Client-owned dogs with confirmed cancer diagnoses were enrolled in FidoCure® by over 200 veterinarians in clinical practice for treatment with small molecule targeted therapy with or without DNA NGS sequencing. A total of 2702 cases enrolled in the FidoCure® Precision Medicine Platform from May 2019 until March 2022. From the total cases, 1108 had formalin-fixed paraffin-embedded (FFPE) tumor tissue available, which were subjected to review to confirm the histological diagnosis. Upon confirmation of cancer by practicing board-certified veterinary pathologists, DNA NGS was performed. The study was reviewed and approved by the One Health Company animal care and ethics committee.

### Next-generation sequencing

The FidoCure® Precision Medicine Platform targets the coding exons of mutated oncogenes and tumor suppressor genes. Genes commonly mutated in human and canine cancers, commercially available in human oncology panels, and targeted by FDA-approved small molecules are prioritized. The following genes were evaluated using an NGS-targeted panel and correlated with treatment and outcomes: *ABL1, ALK, ARID1A, ATM, ATRX, BRAF, BRCA1, BRCA2, CDK4, CDK6, CDKN2A, CREBBP, EGFR, ERBB2, FBXW7, FGFR1, FGFR2, FGFR3, FLT1, FLT3, FLT4, HIF1A, HRAS, IDH1, IDH2, JAK1, JAK2, JAK3, KDR, KIT, KMT2C, KMT2D, KRAS, MAP2K1, MET, MTOR, NRAS, PARP1, PDGFRA, PDGFRB, PIK3CA, PTEN, RB1, RET, SETD2, SMAD4, SMARCA4, TP53*.

Hybrid capture-based enrichment of the targeted genes was performed using the SureSelect custom DNA Target Enrichment Probes and SureSelect XT Hyb and Wash kit following the manufacturer’s instructions. The final library was quantified using qPCR and pooled for sequencing on the Illumina® platform (Illumina, California, USA) with a read length configuration of 150 PE for up to 6 M PE reads (3 M in each direction), yielding an approximate coverage of 612× per sample. Sequencing was performed in a CLIA-certified CAP-accredited laboratory. The sequence read pairs were then mapped to the canine reference genome (CanFam3.1 and CanFam4) using BWA-MEM (v0.7.12)^85^. Mapped read coverage was obtained using GATK (version 3.8.1)^86^ AddOrReplaceReadGroup, MarkDuplicates, RealignerTargetCreator, and IndelRealgner, with minimum mapping quality of 10 and base quality of 10. BamTools^87^ was used to filter out low-quality reads (mapping quality < 5) and a high rate of mismatches (mismatch > 10). As no matching normal samples were used, unique variants were submitted to a pipeline for germline and somatic calling as previously published^17^.

### Dog outcome and dataset

As part of our data collection program, veterinarians had access to targeted therapies (FDA-approved for human treatment) available to prescribe to their patients based on DNA NGS sequencing results. Dogs were provided subsidized access to the following 10 targeted therapies: imatinib, lapatinib, rapamycin (sirolimus), sorafenib, trametinib, vorinostat, dasatinib, toceranib, palbociclib, and olaparib. Cocktails of inhibitors, given orally, were administered to enrolled dogs by their owners at home, typically given daily. Veterinarians were contacted by FidoCure staff to provide clinical updates and outcomes for dogs. Overall survival times were recorded for 2144 of the 2702 total enrolled cases. To better correlate somatic genomic alteration with therapy, germline mutations are excluded from the analysis. We have included the full list of targeted treatments, targeted genes, and cancer types in Supplementary Table 2. Additionally, we have summarized the demographic traits of our panel cohort by age, sex, reproductive status, and weight in Supplementary Table 4.

### Statistical model

We used a Cox proportional-hazards model to fit survival time, implemented using the Lifelines package in Python^88^. The model estimates the risk of death at a given time,

4 $$h\left( {t|X} \right) = h\left( t \right) \ast \exp \left( {X_1\beta _1 + \ldots + X_p\beta _p} \right),$$

where *h* represents the risk of death at time *t*, and *X* represents *p* covariates. Additionally, the ratio of the hazard rates in the treatment group versus the control group is represented as the *hazard ratio*.

### Feature Processing

A one-hot encoded feature for each targeted therapy (treatment) received by the dog was created. For example, if a dog receives multiple treatments, the encoding for each treatment would be a “1”, otherwise, a treatment is encoded as “0”. Similarly, gene mutations were one-hot encoded and treated as independent variables during the analyses. Tumor types were grouped into broad categories to preserve sample size and include them as one-hot encoded features.

Each dog’s survival time and status were determined based on their last known update, which was reported by the veterinarian’s office. A dog is only considered deceased if there is a reported date of death, and is only considered to have survived up until the last known update. Two survival intervals were computed: survival from time of diagnosis and survival from time of treatment. When reporting hazard ratios regardless of intervention, survival time is defined as *survival from time of diagnosis*, which is the time from diagnosis to last known update if the dog is still active, and the time from diagnosis to death if the dog is deceased. When reporting hazard ratios for specific treatments, survival time is defined as *survival from treatment*. The first date of any targeted therapy being ordered is used as the start of the treatment date.

Demographic features, including weight in kilograms, sex, and reproductive status (neutered or spayed vs intact), were extracted and used as covariates in our Cox model. In addition, to control for each dog’s baseline survival rates, the dog’s age at diagnosis, as well as the time from diagnosis to treatment are included, which is computed by subtracting the date that the targeted therapy was first ordered from the date of diagnosis.

### Reporting summary

Further information on research design is available in the Nature Research Reporting Summary linked to this article.

## Supplementary information

Supplementary Section

REPORTING SUMMARY

## Data Availability

We have released aggregate statistics of our data in the paper. De-identified individual level data can be shared with researchers upon reasonable request. The sequencing data for 1064 out of 1108 dogs used in this analysis are available as BAM Files in the NIH SRA archive (SUB12401242), and the remaining 44 are stored in alternate formatting and also available upon request.
