# Supplementary material for: Analyses of canine cancer mutations and treatment outcomes using real-world clinico-genomics data of 2119 dogs
Source: NPJ Precis Oncol. 2023 Jan 19;7:8. doi: 10.1038/s41698-023-00346-3 (PMC9852553; doi:10.1038/s41698-023-00346-3)
Supplement: Supplementary file 1 — Supplementary Section [file 41698_2023_346_MOESM1_ESM.pdf]

Supplementary Section

Supplementary Tables

Hazard Ratios Associated With Gene Mutations in Human Tumors				
**Mutated Gene**	**Hazard Ratio (95% CI)**	**p-value**	**Sample Size (With/Without)**	**Median Survival Days (With/Without)**
TP53	**1.66** (1.60, 1.71)	<0.01	16949/18413	775/1572
PIK3CA	**0.86** (0.82, 0.90)	<0.01	4958/30427	1390/1099
NRAS	**1.09** (0.99, 1.20)	0.07	1101/34312	1045/1139
ATM	**0.93** (0.86, 1.00)	0.05	2064/33329	1459/1119
KIT	**0.73** (0.66, 0.81)	< 0.01	1218/34195	2310/1112

Supplementary Table 1. Hazard ratios associated with mutations in each gene in humans, along with confidence intervals, p-values, sample sizes, and median survival in days. We include only mutations in genes that are found to be statistically significant in canines for purposes of comparison. Sample sizes and median survival time (in days) are reported for humans both with and without mutations in each gene.

Extended Data Details		
**Targeted Treatments**	**Targeted Genes**	**Cancer Types**
Imatinib, Lapatinib, Rapamycin (Sirolimus), Sorafenib, Trametinib, Vorinostat, Dasatinib, Toceranib, Palbociclib, Olaparib	ABL1, ALK, ARID1A, ATM, ATRX, BRAF, BRCA1, BRCA2, CDK4, CDK6, CDKN2A, CREBBP, EGFR, ERBB2, FBXW7, FGFR1, FGFR2, FGFR3, FLT1, FLT3, FLT4, HIF1A, HRAS, IDH1, IDH2, JAK1, JAK2, JAK3, KDR, KIT, KMT2C, KMT2D, KRAS, MAP2K1, MET, MTOR, NRAS, PARP1, PDGFRA, PDGFRB, PIK3CA, PTEN, RB1, RET, SETD2, SMAD4, SMARCA4, TP53	carcinoma - other, pulmonary adenocarcinoma, nasal carcinoma, squamous cell carcinoma, thyroid carcinoma, osteosarcoma, transition cell carcinoma, lymphoma, histiocytic sarcoma, undifferentiated carcinoma, anal sac carcinoma, gi carcinoma, undifferentiated sarcoma, melanoma, mast cell tumor, other, mammary carcinoma, soft tissue sarcoma, hemangiosarcoma, splenic carcinoma

Supplementary Table 2. List of targeted treatments, targeted genes, and cancer types

Hazard Ratios Associated With Gene Mutations				
Mutated Gene	Hazard Ratio (95% CI)	p-value	Sample Size (With/Without)	Median Survival Days (With/Without)
TP53	**1.48** (1.24, 1.77)	<0.01	401/785	220/423
PIK3CA	**1.33** (1.03, 1.72)	0.03	129/1057	189/380
NRAS	**0.61** (0.41, 0.89)	0.01	69/1117	342/359
ATM	**0.48** (0.31, 0.75)	<0.01	59/1127	809/347
KIT	**0.43** (0.21, 0.88)	0.02	31/1155	642/349
ALK	**0.64** (0.42, 0.97)	0.04	68/1118	424/349

Supplementary Table 3. Statistically significant hazard ratios associated with mutation in each gene. We present the same analysis as Table 2, but with the inclusion of germline mutations in the overall analysis (defined as amino acid changes with allele frequencies between 46% and 54% that have more than 5 appearances in our dataset). Hazard ratios are presented with 95% confidence intervals, and only targeted genes with p-values < 0.05 are included. Sample sizes and median survival time (in days) are reported for dogs both with and without mutations in each gene.

Demographic Characteristics of Patient Population	
Trait	Total<br>(n = 2702)
Age at diagnosis - years	9.88 ± 2.76
Sex - no. (%)	
Male	1461 (54.1%)
Female	1241 (45.9%)
Reproductive Status - no. (%)	
Intact	209 (7.7%)
Neutered/Spayed	2489 (3.3%)
Unknown	4 (0.1%)
Weight - kg	25.03 ± 12.87

Supplementary Table 4. Summary statistics for demographics in canine patients. We present the average age (in years) with standard deviation, the percent and number of male and female dogs, the percent and number of intact, neutered/spayed, and unknown status dogs, and the weight in kg with standard deviation.

Dosages for Targeted Treatments in Canines and Humans			
Drug	Canine Dose (mg/Kg)	Human Dose (total)	Human Dose (mg/Kg)
Dasatinib	0.5 - 0.7 mg/Kg qDay[88,**]	100 mg qDay[89]	1.4 mg/Kg qDay
Imatinib	10 mg/Kg qDay[90]	400 - 600 mg qDay[91]	5.7 - 8.5 mg/Kg qDay
Lapatinib	10 - 30 mg/Kg qDay[92]	1250 - 2000 mg dDay[93]	17.8 - 28.5 mg/Kg qDay
Olaparib	3 mg/Kg qDay[94,**]	300 mg q12hr[95]	4.2 mg/Kg q12hr
Palbociclib	0.2 mg/Kg qDay[96,*]	125 mg qDay[97]	1.7 mg/Kg qDay
Rapamycin	0.1 mg/Kg qDay[98]	2 mg qDay[99]	0.02 mg/Kg qDay
Sorafenib	3 - 5 mg/Kg q12hr[100]	400 mg q12hr[101]	5.7 mg/Kg q12hr
Toceranib	2.5 - 3.25 mg/Kg q48hr[102]	Not approved for humans	
Trametinib	0.02 - 0.03 mg/Kg qDay[103,*]	2 mg qDay[104]	0.02 mg/Kg qDay
Vorinostat	15 - 30 mg/Kg qDay[105,**]	400 mg qDay[106]	5.7 mg/Kg qDay

*NOAEL dose or higher based on preclinical studies

**NOAEL dose was decreased based on adverse events reported by veterinarians and pet parents

Supplementary Table 5. Doses used for each targeted treatment in our panel. We present the suggested canine and human drug doses for 10 targeted treatments in mg. In both canine and humans, doses represent clinically suggested doses but may vary in individual cases.

Supplementary Figures

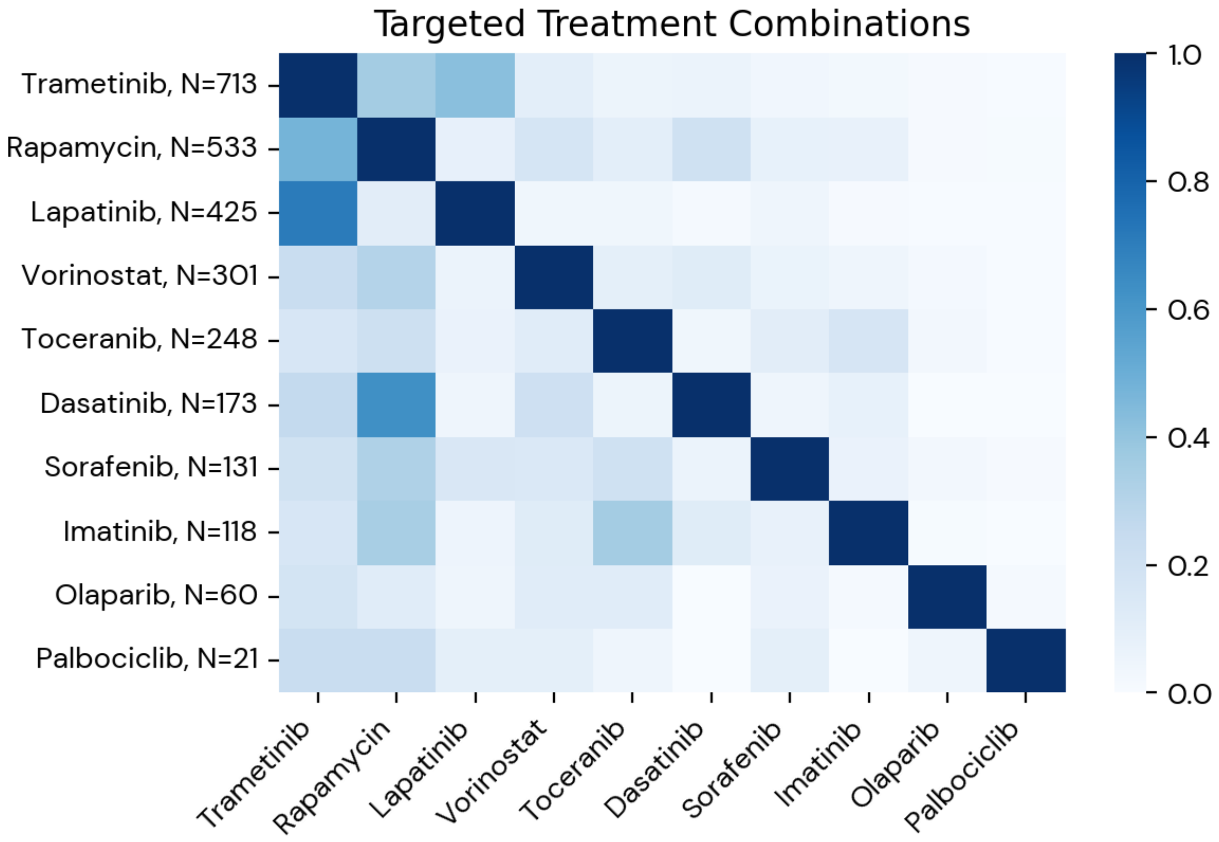

Supplementary Figure 1. Frequency of combinations of targeted therapies used in treatment. Each cell represents the relative frequency of drug co-occurrences, with the number of patients treated with each drug reported as sample size N. Each row represents the relative proportion of patients receiving another drug. For example, a value of 1 would mean that all patients who received a given drug in the row also received the given drug in the column.

Kaplan-Meier Curves for Gene Mutations

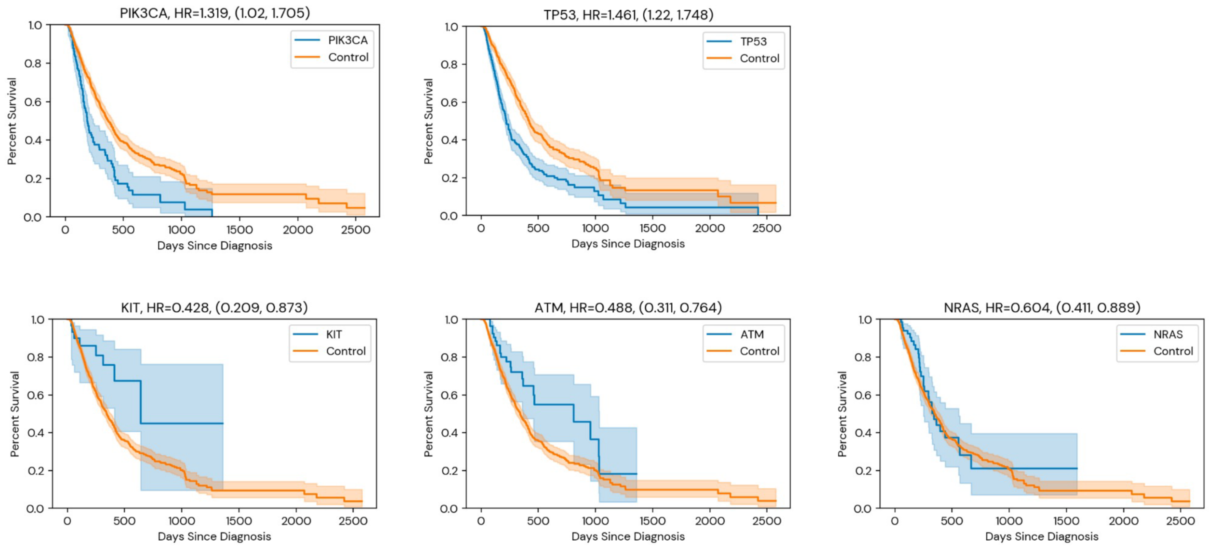

Supplementary Figure 2. Kaplan-Meier curves for gene mutations that are found to be statistically significant to overall survival for canines. In each plot, the x-axis contains the number of days since diagnosis, while the y-axis shows the survival rate at each time point. The blue line represents the given gene's survival curve, while the orange line represents the survival curve for all cases that do not contain that given mutation. Furthermore, the shaded area around each line represents the 95% confidence interval around the risk at each point. At the top of each plot is the name of the gene being analyzed, along with the relative hazard ratio and the 95% confidence interval.

Kaplan-Meier Curves for Tumor Types

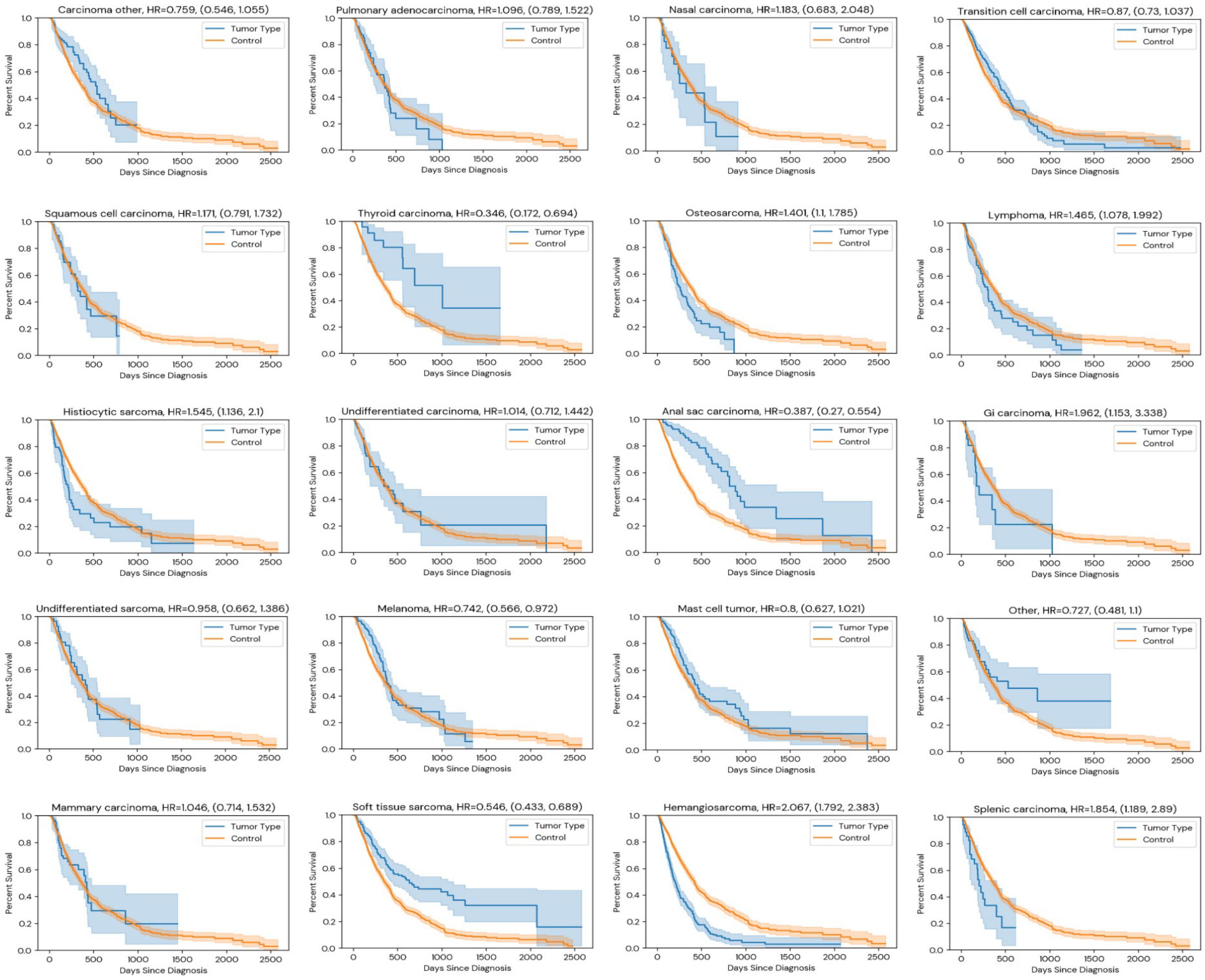

Supplementary Figure 3. Kaplan-Meier curves for tumor types found in our dataset. In each plot, the x-axis contains the number of days since diagnosis, while the y-axis shows the survival rate at each time point. The blue line represents the given tumor type's survival curve, while the orange line represents the survival curve for all cases that do not have the given tumor type. Furthermore, the shaded area around each line represents the 95% confidence interval around the risk at each point. At the top of each plot is the name of the tumor type being analyzed, along with the relative hazard ratio and the 95% confidence interval.

89. Bristol Myers Squibb. Sprycel (dasatinib) [package insert]. U.S. Food and Drug Administration website.https://www.accessdata.fda.gov/drugsatfda_docs/label/2010/021986s7s8lbl.pdf. Revised October 2010. Accessed Oct 11, 2022.

90. Isotani, M. *et al.* Effect of tyrosine kinase inhibition by imatinib mesylate on mast cell tumors in dogs. *J. Vet. Intern. Med.* **22**, 985–988 (2008).

91. Novartis Pharmaceutical Corp. Gleevec (imatinib mesylate) [package insert]. U.S. Food and Drug Administration website.https://www.accessdata.fda.gov/drugsatfda_docs/label/2008/021588s024lbl.pdf. Revised September 2008. Accessed Oct 11, 2022.

92. Maeda, S. *et al.* Lapatinib as first-line treatment for muscle-invasive urothelial carcinoma in dogs. *Sci. Rep.* **12**, 4 (2022).

93. GlaxoSmithKline (GSK). Tykerb (lapatinib) [package insert]. U.S. Food and Drug Administration website.https://www.accessdata.fda.gov/drugsatfda_docs/label/2010/022059s007lbl.pdf. Approved 2007. Accessed Oct 11, 2022.

94. Lynparza (Olaparib); Pharmacology Review. Center for Drug Evaluation and Research, U.S. Food and Drug Administration, (NDA 206162), 01 December 2014.

95. AstraZeneca. Lynparza (olaparib) [package insert]. U.S. Food and Drug Administration website.https://www.accessdata.fda.gov/drugsatfda_docs/label/2020/208558s014lbl.pdf. Revised May 2020. Accessed Oct 11, 2022.

96. Ibrance (Palbociclib); Pharmacology Review. Center for Drug Evaluation and Research, U.S. Food and Drug Administration, (NDA 207103), 22 January 2015.

97. Pfizer. Ibrance (palbociclib) [package insert]. U.S. Food and Drug Administration website.https://www.accessdata.fda.gov/drugsatfda_docs/label/2019/207103s008lbl.pdf Revised April 2019. Accessed Oct 11, 2022.

98. Larson, J. C. *et al.* Pharmacokinetics of orally administered low-dose rapamycin in healthy dogs. *Am. J. Vet. Res.* **77**, 65–71 (2016).

99. Pfizer. Rapamune (sirolimus) [package insert]. U.S. Food and Drug Administration website.https://www.accessdata.fda.gov/drugsatfda_docs/label/2017/021083s059,021110s076lbl.pdf. Revised April 2017. Accessed Oct 11, 2022.

100. Foskett, A. *et al.* Tolerability of oral sorafenib in pet dogs with a diagnosis of cancer. *Vet Med (Auckl)* **8**, 97–102 (2017).

101. Bayer HealthCare Pharmaceuticals Inc. Nexavar (sorafenib) [package insert]. U.S. Food and Drug Administration website.https://www.accessdata.fda.gov/drugsatfda_docs/label/2018/021923s020lbl.pdf. Revised December 2018. Accessed Oct 11, 2022.

102. Bernabe, L. F. *et al.* Evaluation of the adverse event profile and pharmacodynamics of toceranib phosphate administered to dogs with solid tumors at doses below the maximum tolerated dose. *BMC Vet. Res.* **9**, 190 (2013).

103. Mekinist (Trametinib); European Medicines Agency: EMA/CHMP/258608/2014 - Committee for Medicinal Products for Human Use (CHMP) Assessment Report, 25 Apr. 2014.

104. Novartis Pharmaceutical Corp. Mekinist (trametinib) [package insert]. U.S. Food and Drug Administration website.https://www.accessdata.fda.gov/drugsatfda_docs/label/2018/204114s007lbl.pdf Revised

April 2018. Accessed Oct 11, 2022.

105. Zolinza (Vorinostat); Pharmacology Review. Center for Drug Evaluation and Research, U.S. Food and Drug Administration, 13 Sept. 2006.

106. Merck & Co. Zolinza (vorinostat) [package insert]. U.S. Food and Drug Administration website.https://www.accessdata.fda.gov/drugsatfda_docs/label/2018/021991s009lbl.pdf. Revised December 2018. Accessed Oct 11, 2022.
