# Supplementary material for: Analyses of canine cancer mutations and treatment outcomes using real-world clinico-genomics data of 2119 dogs
Source: NPJ Precis Oncol. 2023 Jan 19;7:8. doi: 10.1038/s41698-023-00346-3 (PMC9852553; doi:10.1038/s41698-023-00346-3)
Supplement: Supplementary file 2 — REPORTING SUMMARY [file 41698_2023_346_MOESM2_ESM.pdf]

nature portfolio

Corresponding author(s): NPJPRECISIONONCOLOGY-02381R Kevin Wu

Last updated by author(s): Oct 13, 2022

Reporting Summary

Nature Portfolio wishes to improve the reproducibility of the work that we publish. This form provides structure for consistency and transparency in reporting. For further information on Nature Portfolio policies, see our Editorial Policies and the Editorial Policy Checklist.

Statistics

For all statistical analyses, confirm that the following items are present in the figure legend, table legend, main text, or Methods section.

n/a	Confirmed	
☐	☒	The exact sample size (*n*) for each experimental group/condition, given as a discrete number and unit of measurement
☐	☒	A statement on whether measurements were taken from distinct samples or whether the same sample was measured repeatedly
☐	☒	The statistical test(s) used AND whether they are one- or two-sided<br>*Only common tests should be described solely by name; describe more complex techniques in the Methods section.*
☐	☒	A description of all covariates tested
☐	☒	A description of any assumptions or corrections, such as tests of normality and adjustment for multiple comparisons
☐	☒	A full description of the statistical parameters including central tendency (e.g. means) or other basic estimates (e.g. regression coefficient) AND variation (e.g. standard deviation) or associated estimates of uncertainty (e.g. confidence intervals)
☐	☒	For null hypothesis testing, the test statistic (e.g. *F*, *t*, *r*) with confidence intervals, effect sizes, degrees of freedom and *P* value noted<br>*Give P values as exact values whenever suitable.*
☒	☐	For Bayesian analysis, information on the choice of priors and Markov chain Monte Carlo settings
☒	☐	For hierarchical and complex designs, identification of the appropriate level for tests and full reporting of outcomes
☒	☐	Estimates of effect sizes (e.g. Cohen's *d*, Pearson's *r*), indicating how they were calculated

Our web collection on statistics for biologists contains articles on many of the points above.

Software and code

Policy information about availability of computer code

Data collection	Data were collected in AirTable and processed in the Pandas library in Python.
Data analysis	Analysis was completed in Python 3.9, with Pandas and Scikit-Learn used for data manipulation and the Lifelines package used for survival analysis.

For manuscripts utilizing custom algorithms or software that are central to the research but not yet described in published literature, software must be made available to editors and reviewers. We strongly encourage code deposition in a community repository (e.g. GitHub). See the Nature Portfolio guidelines for submitting code & software for further information.

Data

Policy information about availability of data

All manuscripts must include a data availability statement. This statement should provide the following information, where applicable:

- Accession codes, unique identifiers, or web links for publicly available datasets
- A description of any restrictions on data availability
- For clinical datasets or third party data, please ensure that the statement adheres to our policy

We have released aggregate statistics of our data in the paper. De-identified individual level data can be shared with researchers upon reasonable request.

Human research participants

Policy information about studies involving human research participants and Sex and Gender in Research.

Reporting on sex and gender	N/A, study does not use human data
Population characteristics	N/A, study does not use human data
Recruitment	N/A, study does not use human data
Ethics oversight	N/A, study does not use human data

Note that full information on the approval of the study protocol must also be provided in the manuscript.

Field-specific reporting

Please select the one below that is the best fit for your research. If you are not sure, read the appropriate sections before making your selection.

☒ Life sciences ☐ Behavioural & social sciences ☐ Ecological, evolutionary & environmental sciences

For a reference copy of the document with all sections, see nature.com/documents/nr-reporting-summary-flat.pdf

Life sciences study design

All studies must disclose on these points even when the disclosure is negative.

Sample size	Our study uses panel data collected from veterinary clinics, and our sample size is the full set of cases at the point of data analysis.
Data exclusions	We exclude data from patients whom we do not have full information required to complete the given analysis. For example, in our analysis of gene mutation risks, we exclude patients that do not have complete genomic testing.
Replication	Our observational study comes with statistical uncertainty quantification methods like confidence intervals and p-values.
Randomization	As our study is a retrospective observational study, we address selection biases by controlling for baseline attributes such as demographics, tumor types, and genomic alterations.
Blinding	As part of our retrospective observational study, authors were not blinded regarding cohorts.

Reporting for specific materials, systems and methods

We require information from authors about some types of materials, experimental systems and methods used in many studies. Here, indicate whether each material, system or method listed is relevant to your study. If you are not sure if a list item applies to your research, read the appropriate section before selecting a response.

Materials & experimental systems

n/a	Involved in the study	
☒	☐	Antibodies
☒	☐	Eukaryotic cell lines
☒	☐	Palaeontology and archaeology
☐	☒	Animals and other organisms
☒	☐	Clinical data
☒	☐	Dual use research of concern

Methods

n/a	Involved in the study	
☒	☐	ChIP-seq
☒	☐	Flow cytometry
☒	☐	MRI-based neuroimaging

Animals and other research organisms

Policy information about studies involving animals; ARRIVE guidelines recommended for reporting animal research, and Sex and Gender in Research

Laboratory animals	N/A, no laboratory animals were involved in analysis.

Wild animals	N/A, no wild animals were involved in analysis.
Reporting on sex	N/A
Field-collected samples	N/A, data are observational collected from veterinary clinics and dogs are not subject to experimentation from authors.
Ethics oversight	The study was reviewed and approved by the One Health Company animal care and ethics committee.

Note that full information on the approval of the study protocol must also be provided in the manuscript.
